# Acceptability to and Engagement With a Virtual Sickle Cell Trait Education Program (SCTaware): Single-Center Prospective Study

**DOI:** 10.2196/38780

**Published:** 2022-11-17

**Authors:** Chase M Beeman, Mary Ann Abrams, Kristin N Zajo, Joseph Stanek, Sarah H O'Brien, Peter Chan, Yvette Shen, Ben McCorkle, Latrice Johnson, Deena Chisolm, Toyetta Barnard-Kirk, John D Mahan, Alexandra Martinez-Mendez, Whitney L Phillips, Susan E Creary

**Affiliations:** 1 Center for Child Health Equity and Outcomes Research Nationwide Children's Hospital The Ohio State University Columbus, OH United States; 2 Primary Care Pediatrics Nationwide Children's Hospital Columbus, OH United States; 3 Division of Hematology, Oncology & Bone and Marrow Transplant Nationwide Children's Hospital Columbus, OH United States; 4 Biostatistics Resource Nationwide Children's Hospital Columbus, OH United States; 5 Department of Design The Ohio State University Columbus, OH United States; 6 Department of English The Ohio State University Columbus, OH United States

**Keywords:** virtual education, remote education, internet-based, health education, hematology, patient education, sickle cell, genetic, child, parenting, sickle cell trait, public health education, acceptability, Hemoglobin S-trait, screening, newborn, eHealth, digital health, telemedicine, telehealth

## Abstract

**Background:**

Public health programs are tasked with educating the community on health topics, but it is unclear whether these programs are acceptable to learners. Currently, these programs are delivered via a variety of platforms including in-person, virtually, and over the telephone. Sickle cell trait (SCT) education for parents of children with this trait is one of many education programs provided by the Ohio Department of Health. The novel SCTaware videoconference education program was developed by a research team after central Ohio’s standard program transitioned from in-person to telephone-only education during the COVID-19 pandemic.

**Objective:**

Our objectives were to investigate the acceptability of the format and engagement with the SCTaware education and assess parental worry about having a child with SCT before and after receiving SCTaware.

**Methods:**

This was a single-center, prospective study of English-speaking parents of children <3 years of age identified to have hemoglobin S trait by newborn screening. Parents who *previously* received SCT education by telephone, were able to be contacted, and had access to an electronic device capable of videoconferencing were eligible to complete surveys *after receiving the virtual SCTaware education program*. The SCTaware educator also completed a survey to assess participant engagement. Data were summarized descriptively and a McNemar test was used to compare parental worry before and after receiving SCTaware.

**Results:**

In total, 55 participants completed follow-up surveys after receiving standard SCT telephone education and then completing SCTaware. Most (n=51) participants reported that the SCTaware content and visuals were very easy to understand (n=47) and facilitated conversation with the educator (n=42). All of them said the visuals were respectful and trustworthy, helped them understand content better, and that their questions were addressed. Nearly two-thirds (62%, n=34) reported that the pictures appeared very personal and applied to them. The educator noted most participants (n=45) were engaged and asked questions despite having to manage distractions during their education sessions. Many participants (n=33) reported some level of worry following telephone-only education; this was significantly reduced after receiving SCTaware (*P*<.001).

**Conclusions:**

Our results suggest that SCTaware is acceptable and engaging to parents. While telephone education may make SCT education more accessible, these findings suggest that many parents experience significant worry about their child with SCT after these sessions. A study to evaluate SCTaware’s effectiveness at closing parents’ SCT knowledge gaps is ongoing.

## Introduction

Public health programs are tasked with educating communities on health topics such as diabetes, asthma, and heart disease. The Ohio Department of Health (ODH) oversees many such education programs, the format of which can vary from in-person to telephone to virtual. One example is the sickle cell trait (SCT) education program for parents of newborns identified as having hemoglobin S trait through newborn screening (NBS) [1].

Nearly 3 million people in the United States have SCT, and approximately 2000 infants are born annually with sickle cell disease (SCD) [2], a chronic blood disorder that can lead to pain, stroke, and early mortality [3]. Individuals with SCT are typically asymptomatic, but to make informed reproductive decisions, they must be knowledgeable about their SCT status, SCD, and if their reproductive partner also has SCT. This is pertinent since two parents with SCT have a 25% chance of having a child with SCD and a 50% chance of having a child with SCT. Despite universal NBS that reliably identifies infants with SCT, >80% of individuals of childbearing age with SCT do not know their status [4,5]. This suggests that public health programs that notify and educate parents of infants with SCT are not as well received, accessible, or effective as needed in practice. There have been studies to suggest that using videoconferencing to deliver genetic counseling information to other populations is a method that can increase the level of satisfaction and accessibility to counseling [6], but this format has not been studied in SCT.

The ODH supported one-on-one in-person SCT education of parents of children with SCT by a trained educator prior to 2020, which increased many parents’ knowledge and was well received, but approximately one-third of parents who were eligible to receive this education in central Ohio did not attend these sessions, likely due to difficulties in transportation and access. With the COVID-19 pandemic, ODH shifted to a telephone-only program. While changing to this format has potential benefits of reducing transportation barriers and increasing parental access to SCT education, it also adds challenges, including difficulty building rapport between the educator and parent and the inability to use supporting visual materials to explain SCT. Its effectiveness, acceptability, and impact on parents’ level of worry about having a child with SCT and/or SCD have not been studied. Given work that suggests parents with a trait for a genetic disease report that effective education about their trait decreases their anxiety, increases their preparedness, and increases their sense of control about the potential of having a child with a genetic disease [7], studies are needed to evaluate the effectiveness of novel SCT education programs.

According to Centers for Disease Control and Prevention guidance on providing health education curricula, effective education not only presents clear and understandable information but is also engaging and acceptable to learners in that it addresses their values, attitudes, and beliefs [8]. To overcome some limitations of both in-person and telephone-only education, we developed SCTaware, a health literacy (HL)–informed, videoconferencing-delivered SCT education program. SCTaware allows for at-home access to SCT education with visual materials and the opportunity to build rapport between parent and educator to facilitate engagement, question-asking, and understanding, and to reduce parental worry. Therefore, the aim of this study was to assess the acceptability of the content and format of this program and parental engagement with SCTaware. We also aimed to assess perceptions of worry among parents before and after receiving SCTaware.

## Methods

### SCTaware

SCTaware was developed by a multidisciplinary research team after evaluation of the existing in-person SCT program in central Ohio [9]. SCTaware is a one-on-one videoconferencing education that is delivered by a trained educator to parents of children with SCT identified by NBS. This program focuses on the reproductive implications of having SCT to encourage parents who do not know their SCT status to get tested. It includes having a trained educator provide the content, core knowledge objectives, a plain language–talking guide to support the educator, HL-based communication strategies to increase learner engagement and foster participation (eg, teach-back), and HL-informed and culturally sensitive visuals to support the verbal content [10].

### Study Design

This was a single-center, institutional review board–approved, prospective study of parents of children identified to have hemoglobin S trait by NBS. English-speaking adult biological parents of children <3 years of age with hemoglobin S trait who received telephone education were identified from the telephone educator’s schedule within the electronic mearents who were able to be contacted via telephone by the virtual educator and member of the study team were eligible to participate if they did not have SCD or a child with SCD, had not received SCT education in central Ohio for another child, were not (or partner was not) currently pregnant, and had access to an electronic device capable of videoconferencing

### Study Procedures

Parents who consented to participate were asked to complete surveys after receiving the SCTaware content. In addition to completing a demographics survey and reporting the type of device they used to receive SCTaware, acceptability and level of worry were assessed using the Education Effectiveness Survey (EES). This survey includes an 18-item Likert scale of multiple-choice items and one open-ended item. The EES was developed using a modified version of the education satisfaction survey that was used in a prior SCT study [9]. It was used to evaluate parents’ satisfaction with the virtual educator and visual materials, to assess if SCTaware addressed parents’ learning barriers, to gauge parents’ level of worry (eg, not worried, a little worried, very worried) about having a baby with SCT before and after receiving SCTaware, and to obtain parent input on the best methods to provide additional SCT education after SCTaware. It also allowed participants to provide open-ended comments about their experience.

After each session, the virtual educator also completed a survey to assess parent engagement and distractions, to quantify the number of questions parents asked, to assess parents’ ability to teach-back key content, and to record the time it took to complete the session. The 5-item survey included 4 multiple-choice responses and 1 open-ended question. This survey was pilot-tested with the SCTaware development team. To assess reliability over time, 6 education sessions were also randomly observed by an additional SCTaware development team member who also completed the survey.

### Statistical Analysis

Data were summarized descriptively. Frequency and percentage for qualitative variables and mean or median and IQR were calculated for quantitative variables. A McNemar test was used to compare parental worry before and after receiving SCTaware. A *P* value <.05 was considered statistically significant. Analyses were completed using the base R statistical package (R Foundation for Statistical Computing).

### Ethics Approval

Documentation of verbal informed consent was required prior to study participation (Clinical Trials number: NCT03984500). This study was approved by the Nationwide Children’s Hospital Institutional Review Board (STUDY00000122).

## Results

### Participants

Of the 391 parents of children with SCT who received telephone education between October 2020 and October 2022, 154 (39%) were able to be contacted, and 86 (56%) consented to participate. Of these, 60 (70%) completed the SCTaware education session (Table 1), and 55 (64%) completed the EES.

**Table 1:** Characteristics of participants completing SCTaware education.

Participant characteristic			Participants (N=60), n (%)
Female sex			59 (98)
**Age (years)**			
	18-24	7 (12)	
	25-39	51 (85)	
	40-64	2 (3)	
**Race**			
	Black	46 (77)	
	White	8 (13)	
	Multiracial	5 (8)	
	Other	1 (2)	
**Ethnicity**			
	Not Hispanic or Latino	57 (95)	
	Hispanic or Latino	3 (5)	
**Language spoken at home**			
	English	50 (83)	
	Other	10 (17)	
**Type of electronic device used for education session**			
	Smartphone (Android or iPhone)	37 (62)	
	Computer	14 (23)	
	Tablet or iPad	4 (7)	
	Missing	5 (8)	
**Type of internet connection used for education session**			
	Wi-Fi	47 (78)	
	Data plan	7 (12)	
	Hotspot	1 (2)	
	Missing	5 (8)	

### SCTaware Education

The mean length of the SCTaware education session was 34.4 (median 30, IQR 10) minutes. A total of 14 parents reported having learning barriers (problems hearing, seeing, reading, understanding English, or other) that make it hard for them to learn new things.

### Understandability of Content

Nearly all participants (n=54, 98%) reported the words used by the educator were somewhat to very easy to understand. All participants agreed that they had a chance to ask questions, that these questions were answered, and that they were given the opportunity to teach-back key concepts to ensure that they understood.

All participants agreed or strongly agreed that the SCTaware visuals helped them understand SCT. Most participants (n=47, 85%) reported that the visuals used were very easy to understand, appeared professional (eg, resectful, trustworthy; n=55, 100%), told helpful information (n=42, 76%), and facilitated conversation with the educator (n=42, 76%). Nearly two-thirds (n=34, 62%) reported that the pictures appeared very personal and applied to them, and 27% (n=15) felt that the visuals were actionable, in that they told the participant what to do (eg, directing a participant how to get tested for SCT).

### Education Format Preferences

Most participants either preferred the virtual session (n=29, 53%) or having the combination of the telephone education and then the virtual session (n=21, 38%). A few (n=5, 9%) reported they preferred the telephone-only education to the virtual session. Those who preferred the virtual or the combination of virtual and telephone-only education reported that the virtual format “made it like we (Educator and Parent) were face to face,” and that “it allowed me (Parent) to feel present in the conversation and learning.” They also reported that it allowed the educator “to show them things,” and that “I (Parent) was able to learn at my own pace.” Participants reported their preferences about potential methods to receive additional SCT information after the SCTaware session (Table 2).

**Table 2:** Participant preferences for how to receive additional information on sickle cell trait.

Participant preferences	Participants (n=55), n (%)
A sickle cell trait website on the internet	26 (46)
Another education session by smartphone, computer, tablet, or iPad	25 (45)
Mailed brochure or booklet	17 (30)
Another telephone call	13 (23)
A sickle cell trait mobile app	9 (16)
Video/DVD	3 (5)
Group session	2 (4)
Do not want any more information	5 (9)

### Educator Perceptions on Engagement

The educator reported that most (n=45) of the participants who completed SCTaware were highly engaged in the education; however, 10 did not use their camera on their electronic device, making engagement assessment among these participants challenging. The educator noted that most (82%, n=49) asked at least one question and 40% (n=24) asked more than three questions. The educator also noted that distractions, frequently a young child crying, were common among parents (65%, n=39), but that many distracted participants were still “fairly engaged despite being distracted and made an effort to indicate listening.”

The observers’ and educator’s assessments of perceived engagement aligned for all 6 randomly observed sessions. Perceptions of level of distraction were aligned for 3 sessions and were closely aligned for the other 3 sessions.

### Parent Perceptions of Worry

**Figure 1:**
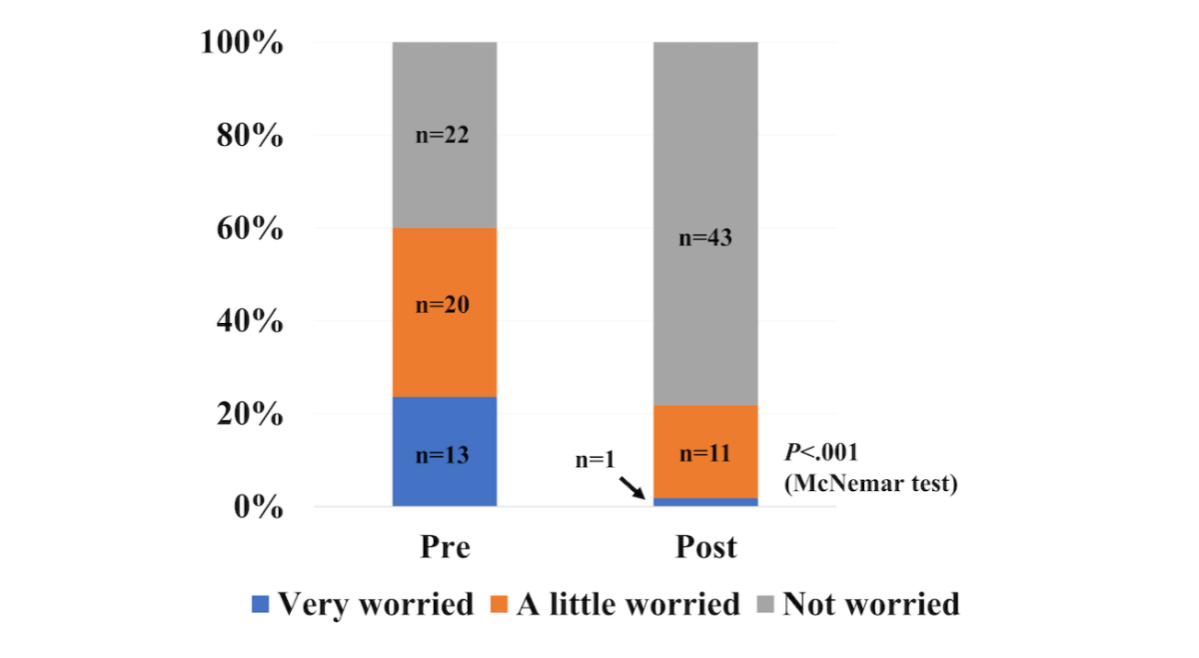
Participants’ self-reported worry about having a child with SCT (Sickle cell trait) before and after SCTaware.

Of the 55 participants who completed the EES, 60% (n=33) reported some level of worry about having a child with SCT after receiving telephone-only education and before receiving SCTaware, 24% (n=13) of those being very worried. After receiving SCTaware, 55% (n=30) of parents had less worry; only 5% (n=3) had increased worry. Only 2% (n=1) remained very worried after receiving SCTaware (Figure 1).

### Participants’ Open-ended Feedback on SCTaware

A few parents reported that the virtual educator “explained things very well” and “got me to think about getting blood work done to see if I'm a trait carrier,” that the education was “very enlightening,” and that “the educator was clear and explained everything in a systematic way that made it easy to understand.” One participant also reported that the teach-back method was “cool...but at times it made me feel like a kid being told to listen up.” However, this same individual also felt that teach-back “helped things to stick a little better [so] it's a thin line between good and unsure.”

## Discussion

### Principal Findings

With an increase in reliance on digital technology use, especially to provide education [11], virtual education has emerged as a method with the potential to close knowledge gaps on important public health topics. Similar studies on genetic counseling provided through videoconferencing services have found that this method of delivery increases accessibility by reducing travel burdens and increasing convenience [6]; however, acceptability of and engagement with these programs among learners in public health education settings has not been extensively studied. This study demonstrated that the SCTaware virtual education program is highly acceptable to parents of young children with SCT. Most virtually educated parents reported that the visuals facilitated understanding of the content and conversation with the educator. This study also suggests that many parents have some level of worry about having a child with SCT after receiving education about it by telephone. This may indicate that telephone-only education is not adequate to fully address parental concerns or may leave parents feeling unable to ask additional questions that may arise after their telephone education session. This may also be because while telephone education is convenient, it may not support learning goals, may not facilitate adequate psychosocial support between educator and learner since educators are not face-to-face with parents, and may inadequately support learning since visual materials cannot be shared. Fortunately, most participants reported a reduction of worry after receiving SCTaware. Additionally, our educator reported high engagement with participants who completed SCTaware, with 82% (n=49) asking at least 1 question, and 40% (n=24) asking more than 3. These findings and parents’ open-ended feedback may reflect the effectiveness of our intentional use of HL strategies, such as putting responsibility on the educator to promote comfort with asking questions and to ensure parent understanding through teach-back. The parents’ positive reports of teach-back underscore its value to confirm understanding. The comment regarding both positive and unsure aspects of teach-back reflects the importance of using it correctly, that is, the educator taking responsibility for being clear [12].

This study found that 62% (n=34) of participants reported the pictures appeared very personal and applied to them. This was lower than expected when compared to the high rating of understandability of visuals by participants. This may reflect efforts to address cultural sensitivity and diversity such that the figures depicted in the visuals had a generic appearance. Additional research is needed to better identify optimal depiction of human figures in SCTaware and similar materials to promote both individual connection as well as inclusiveness. Nevertheless, participants reported high satisfaction, affirming the visuals were very easy to understand, professional, helpful, and facilitated conversation with the educator.

Notably, we observed that participants often faced distractions and interruptions, most of which were out of the parents’ control (eg, a crying child) during their virtual education session. These interruptions did not necessarily impact parental engagement as assessed through the educator survey. However, divided attention while learning has been shown to impair long-term memory retention and decrease one’s ability to apply learned knowledge to new context [13]. This may impact long-term knowledge gain about SCT. A potential strategy to reduce the impact of distractions on knowledge gain could be to ask parents to find a quiet and interruption-free space prior to the education. However, we recognize that this may not always be possible, especially since parents of children with SCT are educated shortly after their child’s birth. Alternatively, encouraging parents to attend to distractions and then re-engaging them may shorten the distraction and allow them to remain attentive to the material when it is being presented.

### Comparison With Prior Work

We observed many parents had high levels of worry after receiving telephone-only SCT education, but this level of concern lessened after SCTaware. This is consistent with prior literature that suggests that effective genetic counseling for abnormal hemoglobin traits can lower anxiety among family members [14]. Future studies that test the effectiveness of virtual education at increasing SCT knowledge and reducing worry, potentially with a follow-up telephone call, are warranted, considering that many parents reported preference for a combination of virtual and telephone education, and nearly a quarter preferred to receive additional SCT information via telephone.

In contrast to our prior study of in-person SCT education [9], where parents rarely asked questions, we found that most parents who received SCTaware asked 1 or more questions. This is notable because question-asking can be used to confirm that learners are engaged with material and is an opportunity for the educator to correct misunderstandings when these may not have been otherwise identified. Furthermore, since parental information-seeking behavior, including collaborative question-asking between parents and genetic counselors, is associated with enhanced knowledge among parents of children with cystic fibrosis [15], this increase in question-asking may ultimately positively impact parents’ SCT knowledge.

### Limitations

This study has a few limitations. First, since only English-speaking parents were recruited via telephone, we were unable to assess SCTaware’s acceptability among those who could not be contacted, were not proficient in English, who did not have the ability to access the virtual education, or who declined participation. It is possible that parents who participated were more motivated, worried, and engaged in learning about SCT and were, therefore, more likely to report high acceptability of SCTaware. While access to the technology required to complete SCTaware could limit the applicability of our program, we suspect this will be less of a limitation with time, since digital technology is becoming more ubiquitous [16]. Development and assessment of acceptable materials to serve parents who do not speak English as a primary language is also an important next step to increasing accessibility to SCTaware. Since genetic counseling sessions that utilize an interpreter have been shown to reduce the number of questions asked by patients and the overall levels of interaction between patients and providers [17], training of future educators and interpreters to ensure thoroughness of education and acceptability by non-English speaking parents is vital, especially since most parents who have children with SCT worldwide may not be proficient in English.

Second, while mothers and fathers were eligible, mothers were primarily recruited. This is likely because mothers were listed as the primary contact for telephone SCT education in the electronic medical record. This finding is consistent with the literature. Studies have found that fewer than a third of fathers attended pretesting cancer genetic counseling appointments, yet findings suggest that attendance of both parents in a genetic counseling session may result in parents feeling more informed [18]. Future research is needed to identify how to reduce this disparity and to investigate if and how mothers communicate their SCT knowledge to their child’s father.

Third, SCTaware was provided by a single educator who was aware that some sessions were going to be randomly observed by members of the study team and evaluated by participants. This may have impacted how the educator provided the education, and future studies need to consider if ongoing evaluation is needed to ensure that the SCTaware program is consistently delivered. Additionally, the acceptability of the virtual program may be related to this educator’s ability to connect with parents and may not be generalizable. Careful selection criteria for future educators and standardized training will be needed to effectively disseminate SCTaware to a larger audience.

Lastly, survey responses may have been biased. For example, participants reported both their pre- and postlevel of worry about having a child with SCT after receiving SCTaware. Postsession impressions may have affected their rating of presession worry. Additionally, participants had an ongoing relationship with the educator as they completed the study activities, which may have influenced their responses. The eucator and participants were also aware when an additional team member observed the education session, which may have impacted the parent’s engagement. Finally, it is necessary to determine if SCTaware closes knowledge gaps and if the program results in increased parental testing.

### Conclusions

Virtual HL-informed SCT education was found to be highly acceptable as a method for distributing information about a common and important public health topic. This suggests that virtual education in the public health setting may be a promising intervention to increase accessibility to other public health information, especially among populations who may have challenges attending in-person education sessions. Future research is needed to determine if this format increases accessibility, whether it closes knowledge gaps and leads to actionable behavior, and if it is applicable to other health topics.

## Abbreviations

EES: education effectiveness survey
HL: health literacy
ODH: Ohio Department of Health
NBS: newborn screening
SCD: sickle cell disease
SCT: sickle cell trait
